# Hair Growth Promoting Effect of *Dicerocaryum senecioides* Phytochemicals

**DOI:** 10.1155/2019/7105834

**Published:** 2019-12-12

**Authors:** H. Rambwawasvika, P. Dzomba, L. Gwatidzo

**Affiliations:** Department of Chemistry, Faculty of Science, Bindura University of Science Education, P. Bag 1020, Bindura, Zimbabwe

## Abstract

Phytochemicals from *Dicerocaryum senecioides* were studied for hair rejuvenation activity using BalB/c mice. Solvent extractions and thin layer chromatography (TLC) were used to extract and isolate the phytochemicals respectively. Phytochemicals were identified by spraying with target-specific revealing reagents. *In vivo* hair growth stimulating activity for each extract was tested on denuded dorsal skin of 5-week old BalB/c mice against the controls and the standard drug minoxidil. The parameters used to evaluate hair growth were hair growth completion time, hair length, hair weight, hair follicle length, and relative hair follicle area. The identified phytochemicals from the active ethanol extract were steroidal glycosides, triterpenoid glycosides, and flavonoid glycosides. Flavonoid glycosides treatment had the uppermost hair rejuvenation capacity as measured by the shortest hair growth completion time (19 days) versus control (29 days) and longest hair length (11.04 mm and 11.86 mm for male and female mice respectively while the control group had 5.15 mm for male mice and 5.33 mm for female mice). Hair growth stimulation by flavonoid glycosides was also dependent on dose concentration. It can be concluded from this study that flavonoid glycosides extracted from the leaves of *Dicerocaryum senecioides* have remarkable hair rejuvenation capacity in BalB/c mice. The present results provides insights on the use of *Dicerocaryum senecioides *for hair rejuvenation in traditional practices and on the potential of the plant as a source of novel compounds that can be used as hair growth promoters.

## 1. Introduction

Hair plays a significant role in people's psychosocial life and anything which negatively affect its appearance will be directly reducing the quality of life for the patients. Hair style is a cultural symbol for expressing one's religion, beauty, wealth, or power, as such involuntary hair loss is a great threat to human social life. Although regarded not life threatening, alopecia is a serious dermatological disorders which can severely affect social status of people. Society has its own perception on people's hair style or lack thereof. The perception can even lead to people diagnosing each other for diseases such as kwashiorkor in children, HIV infection, or cancer in adults [1, 2]. In order to fit in and gain social acceptance, a lot is done to keep one's hair impressive. The extent of this impact can be measured by the fact that every year in the United States of America, around 60 million individuals spend roughly $US1.5 billion on hair regrowth medicines [3]. The complicated process of hair growth constitute 3 cyclic phases which are regeneration phase (anagen), relapse phase (catagen), and rest phase (telogen). A normal healthful scalp will be having an average of 100,000 hairs, 90% of which will be in the rapid growth phase (anagen) at a time.

Hair loss emanate from many factors such as old age, genetic predisposition, thyroid imbalance, undernourishment or wrong diet, chronic infections, hormonal effects of family planning pills, physiological processes such as pregnancy, certain drugs, and chemotherapy targeting cancer cells [4, 5]. In some cases hair loss can be temporary but a hormonal and genetic predisposition condition like androgenic alopecia is usually permanent. Currently such conditions are treated but cannot be cured. In an attempt to counter the deleterious effects of hair loss on the health and self-esteem of people, a wide range of natural, and synthetic products have been tried. Some of the tried products reveal side effects while others lack effectiveness on some patients. This motivates the continuous need to search for new and safer alternatives with improved efficacy. Herbal products have proved over the past years to offer safer and environmentally friendlier medicines compared to synthetic ones and they are worthy trying in the treatment of alopecia.

There are only two approved drugs for alopecia by the Food Drug Administration (FDA), minoxidil (Rogaine), and finasteride (Propecia) [6]. Both drugs are effective however they are not enough to meet the growing need of alopecia remedies both in men and women. Topical minoxidil has generally been considered a safe application with very rare side effects which normally disappear upon stopping medication [7]. Oral finasteride worked effectively against male pattern baldness. Few reports of mild sexual related problems such as irregular ejaculation, lower sperm volume, and poor sexual performance which normally subside upon discontinuation of treatment were observed on clinical trials [8, 9]. Considering that both finasteride (a dihydrotestosterone-suppressing 5a-reductase inhibitor) and minoxidil (an antihypertensive potassium channel opener) are products of serendipity [6], more focus should be given in an attempt to design drugs of specific pharmacological action against hair loss.

Mice hair follicles are synonymous to that of human beings with respect to essential features of organization and function. Several authors established follicular similarities on mice and human beings. Apart from cell type similarities, both follicles experience repetitive cyclic hair growth [10, 11]. Murine are also useful as a preindicator of possible toxicity or irritation of potential medicines. Consequently, preclinical research to understand human hair biology can be done on mice prior to undertaking clinical trials. It is paramount, however, for a researcher to understand hair growth cycle patterns of rodents before selection. Synchronized hair growth in rodents is only when the rodents are young, thereafter numerous hair cycle patterns come into play, each with a different regeneration rhythm [12, 13]. Thus it will be difficult to have all the hair follicles in the same growth phase at any time after the completion of the first hair growth cycle. Physiological events such as lactation and pregnancy can also alter the domain patterns on rodents. This research made use of 5 weeks old BalB/c mice whose dorsal hair was in the late telogen stage of growth to examine the potential of the leaf extract of *Dicerocaryum senecioides *for hair growth stimulation . The same animal model was also used in previous studies [14].

*Dicerocaryum senecioides* is a common herb in Zimbabwean ethnobotany popularly used as a soap substitute, a relish, and to facilitate the removal of trapped placenta in cattle. The herb is also popularly used to stimulate hair growth in alopecia cases although the claim has not yet been established scientifically. A viscous fluid obtained when the herb's leaves are macerated in water is responsible for all the ethnopharmacological uses. The aqueous extract of the herb's conspecific, *Dicerocaryum zangubaricum* was shown to contain many sugars such as galactose, xylose, arabinose, and mannose [15, 16]. Laboratory studies of the plant have also shown to be a reliable source of medicinal antioxidants [17–19]. Studies done by Chokoe [17], Rambwawasvika et al. [20] revealed that the plant extract has antimicrobial properties against some fungi and bacteria. Phytochemical profiling of the leaf extract done by Rambwawasvika et al. [20] revealed the presence of many phytochemicals including phenolic compounds, flavonoids, alkaloids, glycosides, terpenoids, and steroids. In this study active phytochemicals from *Dicerocaryum senecioides* leaf extract were isolated and tested *in vivo* for hair growth stimulation on mice against 2% minoxidil standard and blank controls in an attempt to scientifically test the hair growth stimulation claims of the herbal extracts.

## 2. Materials and Methods

### 2.1. Materials and Chemicals

Standard drug 2% minoxidil supplied by McNeil Products Limited, UK was purchased from a local retail pharmacy. Analytical grade reagents supplied by Merck Germany were used to prepare reagents and solutions. Thin Layer Chromatography plates (ALUGRAM® SIL G/UV_254_) and preparative glass coated TLC plates (60F_254_, 20 × 20 cm) were also supplied by Merck (German).

### 2.2. Plant Material Collection

Wet leaves of the plant *Dicerocaryum senecioides* were harvested in the summer season around Bulawayo city in Zimbabwe. Identification and authentication was done by the Harare Botanical garden and the herb voucher specimens 2017/5 was kept for future reference in the Bindura University of Science Education, chemistry laboratory.

### 2.3. Extraction and Fractionation of Plant Material

The leaves of *Dicerocaryum senecioides* were dried under roof by spreading them on thin sheets of stainless steel in the chemistry laboratory bench tops. The dry leaves were powdered using a laboratory blender. The ground plant leaves (100 g) were extracted exhaustively with absolute ethanol (1000 mL) by shaking for 12 hours on a laboratory shaker. The residue from the extract was removed by filtration using a mutton cloth first and then using Whatman No. 1 filter paper. The extraction process was repeated 3 times with fresh ethanol solvents. The collected supernatants were pooled together for concentration on a rotary evaporator (RE-200) from Xi'an Heb Biotechnology Co., Ltd., China. The solid obtained was resuspended in ethanol: water (60 : 40, v/v) and sonicated to facilitate solubility. The suspension was transferred to a separating funnel and an equal volume of hexane was added followed by a careful thorough shaking. Two fractions, the hexane (D1) and aqueous (D2) were obtained and separated. The extraction process was repeated 3 times with fresh hexane portions before the extracts were concentrated by rotary evaporation. Dried extracts were then kept in amber bottles at 4°C until required for use.

### 2.4. Thin Layer Chromatography (TLC) and Phytochemical Tests

The D1 and D2 extracts were subjected to analytical Thin Layer Chromatography (TLC) for separation and qualitative detection of phytochemicals. Crude extract solutions of 10 mg/mL were prepared by redissolving the extracts in their respective solvents for chromatographic separation. The extracts were spotted on 5 × 10 cm ALUGRAM® Xtra SIL G/UV_254_ TLC plates using a spotting capillary. The plates were then subjected to various solvent systems for separation of phytochemicals. The D1 TLC chromatogram was developed using solvent system hexane; ethyl acetate; acetic acid (HEA, 50 : 40 : 10, v/v/v). The polar fraction (D2) was successfully separated using the solvent system ethyl acetate; methanol; water (EMW, 10 : 2 : 1.5, v/v/v). Detection of flavonoids was done by spraying the TLC plates with 1% AlCl_3_ in ethanol according to the method of [21, 22]. Triterpenoids and steroids were revealed by spraying with Liebermann-Burchard's reagent according to the method adopted from [23, 24].The developed plates were viewed on UV viewer cabin at 366 nm for characteristic florescence and *R*_*f*_ values determined. Glycosides were detected using the method described by [25].

Retention factor (*R*_*f*_) values are calculated using the following formula,

(1) $$\begin{array}{c} R_{f}=\frac{\text{Distance moved by extract}}{\text{Distance moved by solvent}}. \end{array}$$

The extracts were then subjected to preparative TLC for the isolation of identified phytochemicals. Same solvent systems were used to develop the chromatograms on glass backed silica gel coated TLC plates (60F_254_, 20 × 20 cm). The developed plates were then dried under fan to allow all the solvents to evaporate. The plates were developed over a distance of 12 cm using similar solvent systems as used for analytical TLC. Different bands were individually scratched off into clean labelled beakers and redissolved in respective extracting solvents. The contents were thoroughly shaken and the silica removed by centrifugation for 10 minutes at 2500 rpm followed by vacuum filtration with Whatman No. 1 filter paper. The supernatants of the extracts were put in preweighed Petri dishes and the solvent was allowed to evaporate under a fume extractor until a constant solid mass was obtained. Further analytical TLC to check purity was conducted and the dry extracts were kept in amber bottles under refrigeration.

### 2.5. Experimental Animals

Healthy BalB/c mice (18−20 g) were purchased from the department of livestock and veterinary services animal unit section. The conduct and care for animals was approved by the country's division of veterinary services (Department of livestock and veterinary services) for animal research conduct and performed in compliance with guidelines of Bindura University of Science Education's Ethical Research Committee for the use of laboratory animals. The animals were housed at the Astra campus' laboratory in spacious polypropylene cages. Bedding for the animals was made from wood shavings which were replaced every week. Proper ventilation and experimental conditions of an ambient temperature of 25 ± 2°C and 12 hour light/dark cycles were maintained throughout the experimental period. A normal feeding with standard pellet and water was maintained throughout the experiment (*adlibitum*). No experiment was done in the first week to allow the animals to familiarize and adjust to the environment.

### 2.6. Dosage Preparation

Dosages for topical application were made by dissolving the solid isolated phytochemicals in 99% ethanol. A concentration of 200 *µ*g/mL was maintained for each extract except when testing for the effect of concentration dose on hair growth. A small laboratory blender was used to achieve uniform concentrations of extract solutions before treatments.

### 2.7. In Vivo Determination of Hair Growth

**Figure 1:**
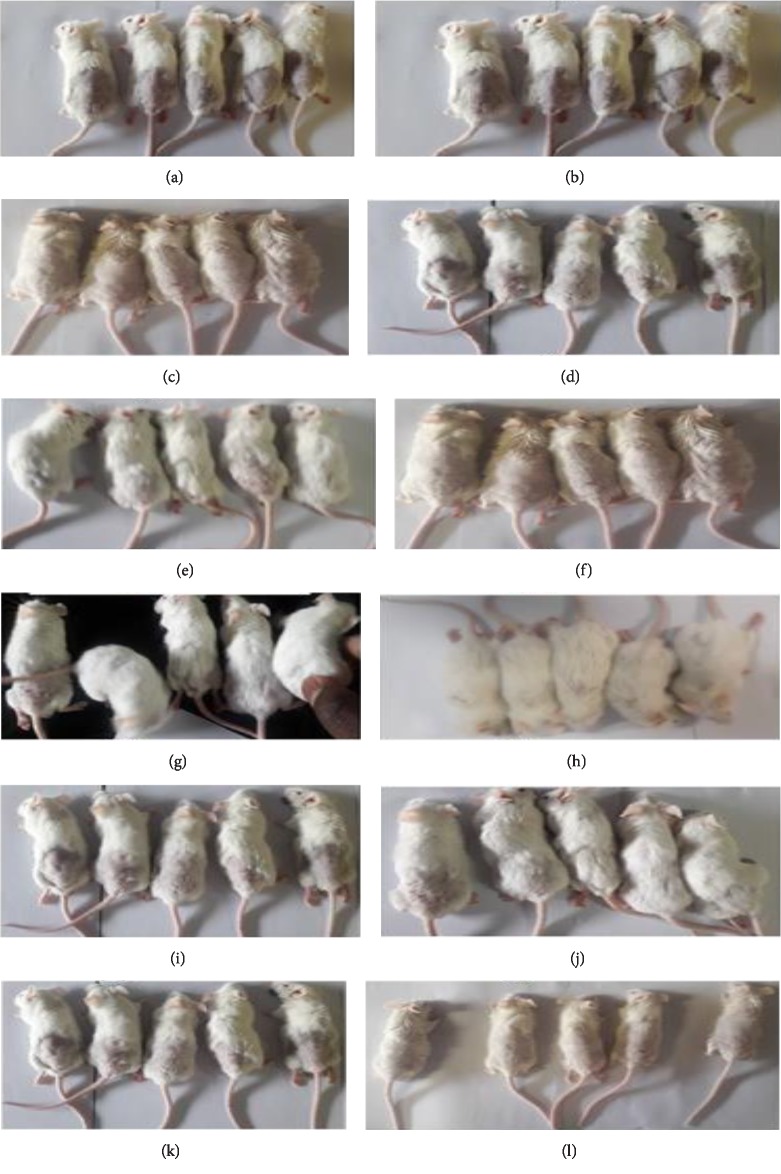
(a and b) Mice at the beginning of the experiment, (c and d) control consisting of ethanol only, (e and f) control consisting of 2% minoxidil, (g and h) experiment consisting of flavonoid glycoside, (i and j) experiment consisting of steroidal glycosides, (k and l) experiment consisting of triterpenoid glycosides after 21 days.

For the experiment, the animals were indiscriminately separated into six groups of (*n* = 10 consisting of 5 males and 5 females, Figure 1) and treated as indicated in Table 1. The mice's dorsal hair covering an area of 4 cm^2^ were shaved using an electric shaver a day before the experiment Figure 1(a). Hair removal cream bought from a local pharmacy was smeared on the shaved portion to completely eliminate all hairs. Special care was taken to avoid damaging the denude skin. Putting the hair growth circle into consideration the tests were topically applied on the 5^th^ week targeting the second telogen phase.

### 2.8. Qualitative Studies on Hair Growth

The minimum time taken before visible hair growth on the shaven skin and the minimum time taken to completely grow new hair on the denude skin were the parameters used for qualitative hair rejuvenation studies. These were achieved by visual observation and the times were noted for each treatment group of mice.

### 2.9. Determination of Hair Length

Hairs were pulled indiscriminately from the previously hairless region of all mice in a group. The average length of the randomly selected 20 hairs was measured and the result noted as the mean length ± standard deviation (SD) of 20 hairs. Length measurements were done after 14 and 21 days of treatment.

### 2.10. Determination of Hair Weight

Hair weight measurements were done at the end of 21 days of treatments. The mice were killed by physical dislocation and a one square centimeter portion of the previously shaved skin region was cut from the same position in all mice. Skin weight with and without hair was determined using an analytical balance. The differences in weight was recorded as the net weight of the new regrown hair.

### 2.11. Determination of Hair Follicle Length and Area

The response of mice follicles to topical application of phytochemicals was done after 21 days of topical application with extracts and controls. New grown hairs were randomly tugged from the once shaved portion of all mice in each group using a pair of high-grip forceps. Hair follicles were viewed and microscopic photographs (magnification 400) were used to determine follicle length. Obtained photographs were used to determine the relative area of follicles. Results were recorded as mean ± SD of 10 strands for each treatment.

### 2.12. Effect of Flavonoid Concentration Dose on Hair Growth

Male mice were put in 6 different groups (5 per group) and their dorsal hair denuded. Each group of mice was subjected to daily topical application of a constant concentration for 21 days. Hair length was measured at day 14 and 21 to determine the response to different concentration doses. The concentrations made were 1000, 500, 200, 100, 50, and 25 *µ*g/mL. Fresh doses were prepared for each application.

### 2.13. Statistical Analysis of Data

Experimental results were stated as mean values ± SD of the mean. Levels of statistical significance were calculated using the Student's *t*-test when comparing values against the control, *P* < 0.05 was considered to be significantly different.

## 3. Results

### 3.1. Phytochemical Analysis

TLC chromatograms of flavonoids glycosides, triterpenoid glycosides, and steroidal glycosides were seen as blue, brown, and red luminous zones in the long wavelength before spraying with revealing agents. Spraying with revealing reagents enabled the identification of the phytochemicals and the subsequent calculation of their *R*_*f*_ values (Table 2).

**Figure 2:**
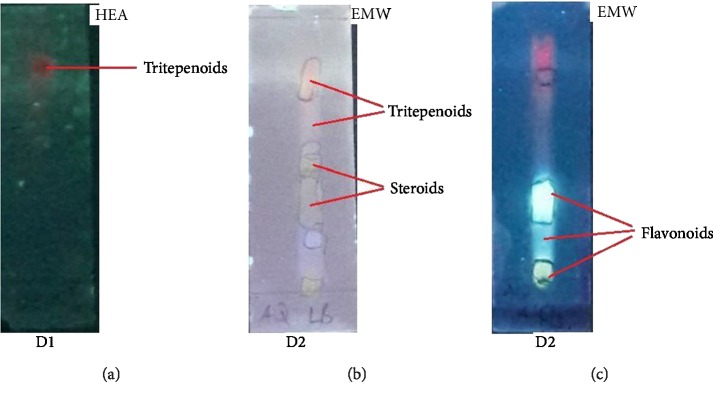
Analytical TLC chromatograms for the two fractions D1 (nonpolar) developed by solvent system HEA and D2 (polar) developed by solvent systems EMW after spraying with revealing agents for the detection of phytochemicals.

Phytochemical analysis revealed the presents of steroidal glycosides, triterpenoid glycosides, and flavonoid glycosides as the major constituents in the ethanol extract of *Dicerocaryum senecioides* leaves. Hexane extract (D1) had one major compound which was shown to be a triterpenoid glycoside while the polar extract (D2) revealed the presence of all the 3 phytochemicals (Figure 2).

### 3.2. Qualitative Evaluation of In Vivo Hair Growth

The minimum time taken before visible hair growth on the shaven skin and the minimum time taken to completely grow new hair on the denude skin were observed physically and selected results are shown in Figures 1(a)–1(l). Treatment with flavonoid glycosides resulted in the shortest hair growth starting time and hair growth completion time (Table 3 and Figures 1(g) and 1(h)). Treatment with flavonoid glycosides reduced the time for hair growth completion by 10 days while the standard 2% minoxidil (Figures 1(e) and 1(f)) reduced the duration by 7 days. The initial time taken for noticeable hair regrowth was reduced by 3 days for both 2% minoxidil and flavonoid glycosides.

### 3.3. Determination of Hair Length

**Figure 3:**
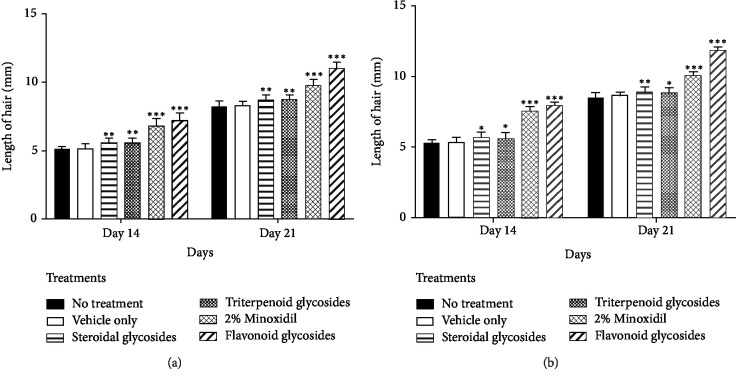
Length of hair on experimental groups after 14 and 21 days of treatment with extracts and 2% minoxidil drug ((a) male; (b) female). Results are shown as mean values ± SD. ^∗^*P* < 0.05, ^∗∗^*P* < 0.01, ^∗∗∗^*P* < 0.001 compared with no treatment control group.

Mice treated with flavonoid glycosides extract showed the longest length of 11.70 ± 0.24 mm for male mice and 11.81 ± 0.23 mm for female mice after 21 days of treatment (Figure 3). The performance was higher than both the controls and the standard drug (2% standard minoxidil). Formulations with phytochemicals steroidal glycosides and terpenoid glycosides treatments had their lengths above the controls but less than that of 2% minoxidil.

### 3.4. Determination of Hair Weight

**Figure 4:**
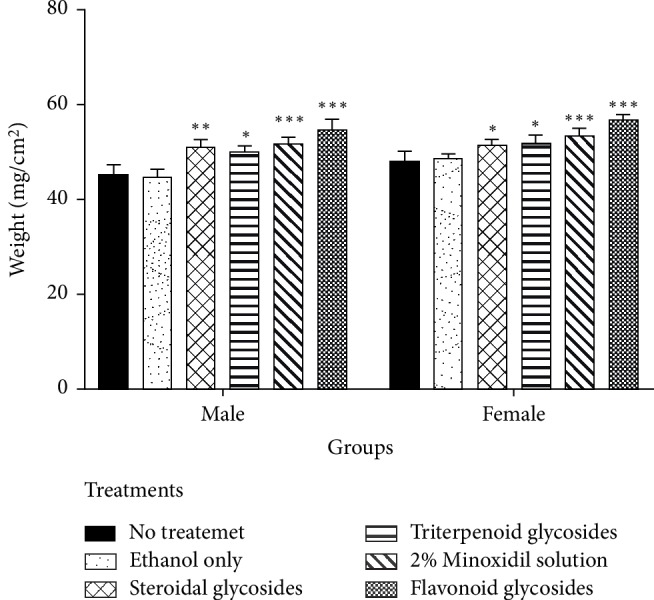
Hair weight measurements after treatment with phytochemical extracts of *Dicerocaryum senecioides*, controls, and 2% minoxidil after 21 days of treatment. Results are graphed as mean values ± SD. ^∗^*P* < 0.05, ^∗∗^*P* < 0.01, ^∗∗∗^*P* < 0.001 compared with no treatment control group (*n* = 10).

As with length, the weight of hair from the mice treated with flavonoid glycoside extract was more than the weight of both controls and the standard. Mice treated with flavonoid glycosides extract had hair weight of 56.61 ± 1.30 mg and 54.44 ± 2.52 mg for male and female mice respectively while those treated with standard minoxidil drug had a weight of 53.34 ± 1.72 mg and 51.81 ± 1.36 mg for male and female mice respectively. Groups treated with steroidal glycosides and triterpenoid glycosides had hair weight less than that of the standard drug (Figure 4).

### 3.5. Effect of Application on Follicle Length, Width, and Relative Area

**Figure 5:**
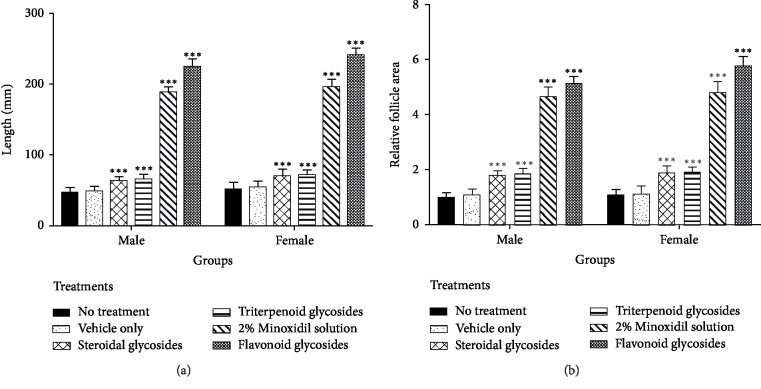
Hair follicle response to different treatments. (a) Hair follicle length measurements, (b) estimated relative follicle area. Results are graphed as mean values ± SD.

**Figure 6:**
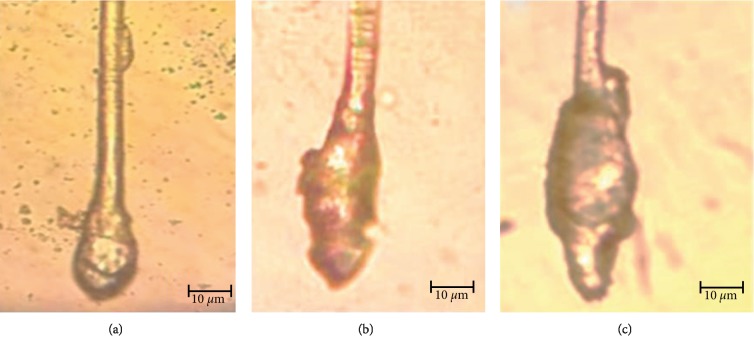
Microscopic images of hair follicles at a magnification of ×400. (a) Image of control untreated, (b) treated with standard 2% minoxidil drug, and (c) treated with flavonoid glycosides extract.

The lengths and widths of follicles were greatest for the mice treated with flavonoid glycosides further identifying flavonoid glycosides as the most bioactive phytochemicals. Images of follicles from the treated mice were used to estimate the area of the hair follicles relative to the controls (Figure 5). Sampled microscope photographs are shown in Figure 6. The obtained results confirmed that the flavonoid glycosides extract as the most effective in causing hair growth as measured by follicle area. The performance of flavonoid glycoside extract was higher than that of the control drug minoxidil while that of steroidal glycosides and triterpenoid glycosides were lower (Table 4). No difference was observed on the performance of steroidal glycosides and triterpenoid glycosides.

### 3.6. Effect of Flavonoid Concentration Dose on Hair Growth

**Figure 7:**
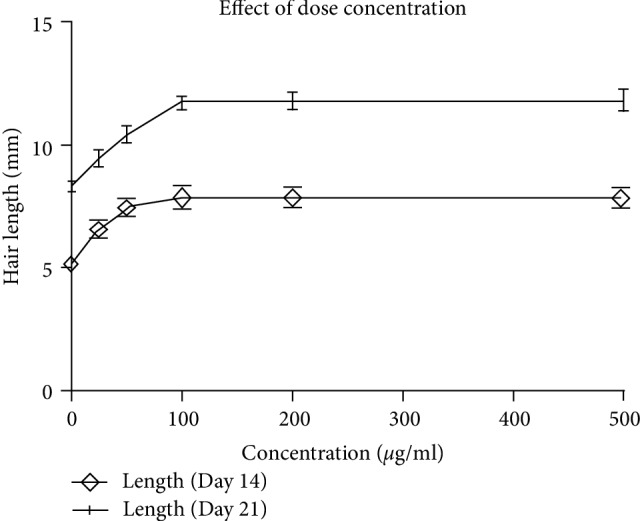
Effect of flavonoid glycoside concentration on mice hair length. Hair length was measured at day 14 and day 21 of daily topical application with flavonoid glycoside extract.

The results of varied concentrations on hair growth at 14 and 21 days indicated that mice hair growth was dependent on the concentration of flavonoid glycosides. An increase in dose concentration to 100 *µ*g/mL was accompanied by an increase in hair length (Figure 7). A further increase concentration above 100 *µ*g/mL did not result in a further increase in hair length.

### 3.7. Toxicity Studies

Although *D. senecioides* leaves are a common relish, investigations to determine the toxicity on mice were done before the experiment commenced. A concentration of 200 mg/mL of crude extract were applied on shaved mice and visual observations were made for any lethal reactions or erythema on skin surface for a total period of 72 hours after the topical applications. The observations warranted ethanol extracts of *D. senecioides* as safe for topical application.

## 4. Discussion

Extractable phytochemicals have proven to be an excellent source of medication for many ailments. Plant extract derived medication is usually associated with fewer side effects in the body and lower production cost [26]. In this study, flavonoid glycosides extracted from *Dicerocaryum senecioides* have proven to be a strong hair growth stimulant when tested *in vivo* on BalB/c mice. Results obtained after 21 days of topical application with separated phytochemicals show significant hair growth in the group of mice treated with flavonoid glycosides compared to other phytochemicals as well as blank control (*P* < 0.01) Figures 1(a)–1(l). The performance of flavonoid glycosides was also significantly greater than that of the standard drug minoxidil (Figures 3–5). Apart from a visible increased hair length above the controls and the standard drug (Figure 3), the group of mice treated with flavonoid glycosides had heavier hair (Figure 4) as well as the biggest follicle size (Figures 5 and 6) after 21 days of receiving treatment. Hair growth was fast in female mice compared to their male counterparts basing on the measured parameters.

If the diverse forms of alopecia are to be put into consideration, flavonoid glycoside extract has potential therapeutic effect on nonhormonal forms such as chemotherapy induced alopecia, traction alopecia, anagen effluvium, telogen effluvium, and some forms of nonhormonal alopecia areata. The extract is yet to be tested for the treatment of androgenic alopecia as the mice model used for tests in this study did not address the condition. Androgenic alopecia results from a combination of genetic predisposition and an androgen called dihydrotestosterone generated from the metabolism of testosterone [8]. The condition affects both male and female but affect males mostly because of the high levels of testosterone in males. Testing for the extract's effect on androgenic alopecia will require other animal models like stump tailed macaque which develop similar scalp baldness due to the generation of androgens in its body [9, 27].

Positive identification of flavonoids on TLC was done by spraying with ethanolic aluminum chloride reagent. A sparkling bluish to yellow color characterized flavonoids (Figure 2). The observed chromatograms were correlating with the findings of [28]. Other prominent phytochemicals, steroidal glycosides, and triterpenoid glycosides in the ethanol extract also significantly stimulate hair growth when compared to the negative controls however their performance was significantly lower than that of flavonoid glycosides and the standard drug minoxidil (*P* < 0.05). Further studies are needed to establish whether their structure does not contain part of the active phytochemical found abundantly in flavonoid glycosides. There was no major difference between the blank control group and the ethanol only group indicating that the extracting solvent has no influence in the physiology of hair growth.

Following the establishment that mice hair cycle is synonymous to that of human with the exception of specialized follicles and a shorter hair growth cycle [29], BalB/c mice were chosen for this study. The same type of mice were also used by [14] in a similar study. Treatment on mice was commenced at the age of 5 weeks to target the late telogen stage were hair follicles are miniaturized and therefore cannot actively grow. Thus any positive hair growth on denuded mice is less attributed to the natural mice hair growth cycle but the treatment which triggers a transformation into the anagen, which is the active hair growth phase. After the first treatment, no mice were not subjected to the second treatment because the second hair growth in mice is not synchronized as not all hairs will be in the same phase of growth [12]. Physiological processes such as pregnancy and lactation were prevented during the experimental period by keeping the male and female mice in separate cages. Physiological processes such as pregnancy and lactation are known to affect the synchronized hair growth patterns [30].

Although mechanistic studies of how flavonoid glycosides in *D. senecioides* promote hair growth are still underway, it is important to discuss how other similar extracts have worked to promote hair growth. Studies on *D. senecioides* extracts, [17–19] investigated the anti-inflammatory and antioxidant properties of the plant extracts. These properties are crucial in triggering proliferation of hair follicle cells leading to hair growth. Studies by [31] on hair growth promoting effects of antioxidants and anti-inflammatory extracts of *Rosemarinus officinalis* and *Altheae officinalis* supported the idea. The mechanism involves follicle stem cells resuscitation by the cleansing removal of microinflammations which emerge from stress and exposure to free radicals. Removal of microinflammations results in cell viability and multiplication of follicle cells. Having flavonoids as active hair growth stimulant is a double blessing in that they are also very good antioxidants. Antioxidants have been incorporated into cosmetic formulations for the intention to lessen the unfavorable impacts of ultra violet (UV) radiation on hair fiber. Studies done by [32] revealed that UV light damage hair development by targeting melanin pigment and protein fractions.

This is not the first time when flavonoids from plant extracts are shown to promote hair growth. Studies done by [33, 34] on *Ginkgo biloba, *a plant known for its hair growth activity was also shown to have flavonol glycosides phytochemicals in larger proportion. Antioxidants like flavonoids are known to stimulate hair growth by causing muscle relaxation in blood vessels around hair follicles thereby facilitating a constant supply of blood with nutrients to the hair follicles cells. A conducive environment for hair regrowth is set by providing decent food nutrients without toxins. On the off chance that these prerequisites are not satisfied, the follicles stays in the dormant resting stage of the hair cycle. The activity of these flavonoid glycosides can also be attributed to their saponification value where the cleansing action is responsible for sloughing of dead skin leading to the opening of scalp pores. This stimulates the hair root and accelerates the conversion of the telogen to anagen stage of hair development. This will enable blood capillaries carrying nutrients and oxygen to reach follicle cells without much hindrances. Research by [35, 36]. suggest that flavonoids can also stimulate hair growth by inhibiting the activity of type II 5*α* reductase enzyme.

## 5. Conclusion

Flavonoid glycosides extracted from the leaves *of Dicerocaryum senecioides* have exhibited remarkable hair growth stimulation in mice *in vivo*. Its hair growth activity was found to be greater than other phytochemicals and that of the standard drug, 2% minoxidil. The extract facilitated anagen induction in telogen follicles of BalB/c mice. The results indicated that flavonoid glycosides from the plant are a promising source of lead compounds for alopecia medication. The activity of the extract on mice was shown to increase with an increase in concentration dose.

## Figures and Tables

**Table 1:** Groups of rats for the experiment.

Group	Treatment
1	Shaved and no chemical applied
2	Shaved and treated with ethanol only
3	Shaved and treated with standard 1 mL of 2% minoxidil ethanolic solution
4	Shaved and treated with vehicle + flavonoid glycosides
5	Shaved and treated with vehicle + steroidal glycosides
6	Shaved and treated with vehicle + triterpenoid glycosides

**Table 2:** Identity of compounds, calculated *R*_*f*_ values, and the systemic mobile phases used on TLC.

Compound	Extract	*R*_*f*_ values	TLC mobile phase
Flavonoid glycosides	D2	0.125	EMW, 10 : 2 : 0.5, (v/v/v)
	D2	0.313	
	D2	0.375	
			
Steroidal glycosides	D2	0.425	EMW, 10 : 2 : 0.5, (v/v/v)
	D2	0.500	
			
Triterpenoids glycosides	D2	0.650	EMW, 10 : 2 : 0.5, (v/v/v)
	D2	0.850	
	D1	0.875	HEA, 50 : 40 : 10, (v/v/v)

**Table 3:** Hair growth initiation and completion time of mice after receiving different treatments.

	No treatment	Vehicle only	Steroidal glycosides	Triterpenoid glycosides	Flavonoid glycosides	Minoxidil
Hair growth initiation/day	8	8	6	6	5	5
Hair growth completion time/day	29	29	24	23	19	21

**Table 4:** Effect of application on relative hair follicle area.

Group	Treatment administered to animals	Relative area of hair follicle	
		Male	Female
A	No treatment	1 ± 0.15	1.08 ± 0.20
B	Ethanol only	1.10 ± 0.20	1.12 ± 0.28
C	2% Minoxidil solution	3.84 ± 0.29^∗∗∗^	3.96 ± 0.33^∗∗∗^
D	Flavonoid glycosides	4.25 ± 0.18^∗∗∗^	4.75 ± 0.29^∗∗∗^
E	Steroidal glycosides	1.80 ± 0.16^∗∗∗^	1.89 ± 0.22^∗∗∗^
F	Triterpenoid glycosides	1.87 ± 0.18^∗∗∗^	1.93 ± 0.17^∗∗∗^

Relative area of hair follicles compared to the standard non treatment group. Results are tabulated as mean values ± SD.^∗^*P* < 0.05, ^∗∗^*P* < 0.01, ^∗∗∗^*P* < 0.001 compared with no treatment control group (*n* = 10).

## Data Availability

The data used to support the findings of this study are included within the article.
